# PBX3 Promotes Oligodendrocyte Progenitor Cell Proliferation and Differentiation During Early Postnatal Development

**DOI:** 10.3390/jdb14030042

**Published:** 2026-09-08

**Authors:** Yanling Yang, Jing Ling, Zhongzhe Zhang, Jie Yuan, Kaixiang Zhang, Xiang Xiao, Hui Guo, Ming Zhang, Xianghui Zhao

**Affiliations:** 1 Yan’an Medical College, Yan’an University, Yan’an 716000, China; 2 Department of Neurobiology, School of Basic Medicine, Fourth Military Medical University, Xi’an 710032, China; 3 School of Life Sciences, Central South University, Changsha 410083, China; 4 The Shaanxi Province Key Laboratory of Brain Function Analysis and Modulation, Xi’an 710032, China

**Keywords:** PBX3, oligodendrocyte, development, myelination

## Abstract

Pre-B-cell leukemia transcription factor 3 (PBX3) is a TALE homeodomain protein involved in neural development, but its function in oligodendrocyte (OL) differentiation has remained unexplored. Here, we show that PBX3 expression is dynamically regulated during OL lineage progression, with peak expression in the corpus callosum at P14, corresponding to the onset of active myelination. In cultured primary oligodendrocyte precursor cells (OPCs), PBX3 expression peaked at OL-1DIV, an early stage of in vitro differentiation. Using OL lineage-specific *Pbx3* conditional knockout mice, we found that *Pbx3* deletion reduced the density of SOX10^+^ OL lineage cells and CC1^+^ mature OLs in the corpus callosum at P14 and P27, accompanied by decreased MBP expression. Notably, these deficits were largely resolved by P50, indicating an age-dependent rather than absolute requirement for PBX3. EdU labeling revealed reduced OPC proliferation in *Pbx3* cKO mice, though the *Olig1-Cre* driver used for conditional deletion is active in early neural progenitors, and thus contributions from reduced OPC specification or generation cannot be excluded. Consistently, siRNA-mediated *Pbx3* knockdown in primary OPCs impaired differentiation and reduced MBP expression in vitro. Collectively, our findings identify PBX3 as a positive regulator of OL differentiation during early postnatal development, with a temporally restricted requirement, and provide insights into the transcriptional control of CNS myelination.

## 1. Introduction

Myelination is a fundamental process in central nervous system (CNS) development, wherein oligodendrocytes generate myelin sheaths that ensheath axons to facilitate rapid saltatory conduction and provide metabolic support to neurons. This process depends critically on the precisely orchestrated differentiation of oligodendrocyte precursor cells (OPCs) into mature myelinating oligodendrocytes, a sequence of events that continues postnatally and is essential for normal neurological function [1]. Disruption of oligodendrocyte differentiation and myelination has been implicated in a range of neurological disorders, including periventricular leukomalacia, multiple sclerosis, and white matter injury following cerebral ischemia [2].

The transcriptional regulation of oligodendrocyte differentiation has been extensively investigated, revealing a hierarchical network of transcription factors that coordinate lineage progression. Key regulators such as OLIG1, OLIG2, SOX10, MYRF, and NKX2.2 have been established as critical determinants of oligodendrocyte specification and maturation [3]. However, despite these advances, the complete repertoire of transcriptional regulators governing the transition from OPCs to mature oligodendrocytes remains incompletely defined, particularly regarding the roles of homeobox-containing transcription factors.

The PBX (Pre-B-cell leukemia homeobox) family of transcription factors, including PBX1, PBX2, and PBX3, are TALE (three-amino acid loop extension) class homeodomain proteins that function as essential co-factors for Hox proteins and other transcription factors in regulating developmental processes [4,5,6]. Among these family members, PBX3 has been relatively understudied in the context of CNS development, particularly regarding oligodendrocyte biology. Recent evidence has begun to implicate PBX3 in neural cell regulation. Using single-cell transcriptomic analysis, Li and colleagues identified PBX3 as a significantly enriched gene in the context of ischemic brain injury, where it participates in the PBX3/Dguok/Kif21b signaling pathway that mitigates OPC dysfunction following middle cerebral artery occlusion [7]. This finding suggests a potential role for PBX3 in OPC responses to pathological conditions, yet its physiological function in oligodendrocyte differentiation during normal CNS development remains unexplored.

In this study, we analyzed the expression dynamics of PBX3 during distinct stages of oligodendrocyte differentiation. Furthermore, we investigated the role of PBX3 in oligodendrocyte differentiation using a combination of in vivo and in vitro approaches. We generated conditional *Pbx3* knockout mice and examined the progression of oligodendrocyte differentiation at multiple postnatal time points. Additionally, we performed siRNA-mediated knockdown of *Pbx3* in cultured OPCs to assess its effects on differentiation. Our findings demonstrate that PBX3 deficiency suppresses oligodendrocyte differentiation both in vivo and in vitro, identifying PBX3 as a novel positive regulator of oligodendrocyte maturation. These results expand our understanding of the transcriptional network controlling myelination and may have implications for remyelination strategies in demyelinating diseases.

## 2. Materials and Methods

### 2.1. Animal

All animal experiments were performed according to the National Institutes of Health Guidelines on the Use of Laboratory Animals and approved by the Animal Care and Use Committee of Fourth Military Medical University. *Pbx3* ^flox/flox^ mice were generated by Cyagen Bioscience (Suzhou, China) and crossed with *Olig1*-*Cre* [8] mice to generate *Pbx3* cKO (*Pbx3* ^flox/flox^; *Olig1-Cre*^+/−^) offsprings. To minimize pain, suffering, and distress, mice were housed in standard cages with ad libitum access to food and water, under 12-hour (h) light/dark cycles with controlled temperature and humidity. Gentle handling was practiced during all procedures. No invasive surgical interventions were performed in this study. Animals were monitored daily for health status, and no unexpected mortality or severe distress was observed throughout the experiments. No animals met humane endpoints during the experiments.

### 2.2. OPC Culture and Immunocytochemistry

Mouse OPCs were isolated from the cortices of pups at P6 as described previously [9]. Briefly, cerebral hemispheres from different pups were pooled, diced, and digested with papain at 37 °C. Following gentle trituration, cells were resuspended in a panning buffer containing insulin (5 μg/mL) and then incubated at room temperature sequentially on three immunopanning dishes: RAN-2 (ATCC #TIB-119, 1:5 hybridoma supernatant), anti-GalC (1:5 hybridoma supernatant), and O4 (1:5 hybridoma supernatant). O4^+^ GalC^−^ OPCs were released from the final panning dish with trypsin (Sigma-Aldrich, St. Louis, MO, USA). OPCs were cultured in Sato medium with PDGF-AA (10 ng/mL, Thermo Fisher Scientific, Waltham, MA, USA, 100-13A) and bFGF (10 ng/mL, Thermo Fisher Scientific, Waltham, MA, USA, 100-18B), at a density of 10^5^ cells/cm^2^. CNTF (10 ng/mL, Thermo Fisher Scientific, Waltham, MA, USA, 450-13), NAC (5 μg/mL, Sigma-Aldrich, St. Louis, MO, USA, A8199), and T3 (40 ng/mL, Sigma-Aldrich, St. Louis, MO, USA, T2877) were added to the Sato medium for oligodendrocyte differentiation. The purity of OPCs and mature oligodendrocytes has been assessed in our previous publication [10].

For immunocytochemistry, cell cultures were fixed in 4% paraformaldehyde (PFA). After Triton X-100 permeabilization for 15 min, samples were incubated with primary antibody for 1 h at room temperature (RT), followed by the fluorescent secondary antibody for another hour. Cells were then counterstained with DAPI (4’,6-diamidino-2-phenylindole) and visualized with a confocal microscope. Experiments were replicated using cells from three different primary cultures.

### 2.3. Duplex siRNA Transfection

For in vitro *Pbx3* knockdown, purified OPCs were transfected with 50 nM duplex siRNA against *Pbx3* (siRNA-307: sense, 5’ GCUCUGAACUGUCACAGAATT 3’; anti-sense, 5’ UUCUGUGACAGUUCAGAGCTT 3’; siRNA-758: sense, 5’ GCACGUGUGAAGCAGUUAUTT 3’; anti-sense, 5’ AUAACUGCUUCACACGUGCTT 3’; siRNA-1230: sense, 5’ CCUCCGUCAUGUUAUCAAUTT 3’; anti-sense, 5’ AUUGAUAACAUGACGGAGGTT 3’) or control nontargeting siRNA (Genepharma, Shanghai, China) using Lipofectamine RNAiMAX (Thermo Fisher Scientific, Waltham, MA, USA, 13778100). Six hours after transfection, the cultures were changed to a differentiation medium. Two or three days later, cultures were harvested for quantitative real-time PCR (qRT-PCR) assay or immunocytochemistry as indicated.

### 2.4. Quantitative Real-Time PCR

Total RNA was purified from tissues or cell cultures using TRIzol reagent according to the manufacturer’s instructions (Thermo Fisher Scientific, Waltham, MA, USA, 15596026CN). For qRT-PCR, RNA was transcribed to cDNA with the PrimeScript™ RT reagent Kit (Perfect Real Time, Takara, Tokyo, Japan, RR037A), and reactions were conducted with LightCycler^®^ RNA Master SYBR Green I (Roche, Basel, Switzerland, 03064760001) in CFX96 Touch Real-Time PCR Detection System (Bio-Rad, Hercules, CA, USA). Relative gene expression was normalized to the internal control β-actin. Primer sequences for target genes are: *Pbx3* (forward primer 5’-CGAGGCGCAAGCAAAGAAAC-3’, reverse primer 5’-TGCCAAAAGCATATTGTCCAGT-3’); *Mbp* (forward primer 5’-GGGATCGTGCAAGGCTTCTT-3’, reverse primer 5’-GGCACCCATTTAGGAGCATTAT-3’); β-actin (forward primer 5’-GGCTGTATTCCCCTCCATCG-3’, reverse primer 5’-CCAGTTGGTAACAATGCCATGT-3’).

### 2.5. Western Blot Analysis

For the Western blot assay, whole-cell lysates were prepared from tissue or cultures using RIPA buffer (150 mM NaCl, 50 mM Tris-HCl (pH 7.4), 1% NP40, 1 mM PMSF, 1× Roche complete mini protease inhibitor cocktail, and 1× Pierce phosphatase-inhibitor cocktail). Protein concentrations in centrifugation-clarified cell lysates were measured using the BCA Protein Assay Kit (Thermo Fisher Scientific, Waltham, MA, USA, A65453), and equal amounts of protein (10 μg) were separated on an SDS-PAGE gel and transferred to Hybond PVDF (Amersham Biosciences, Marlborough, MA, USA, 10600029). The following primary antibodies were used: PBX3 (Novusbio., Littleton, CO, USA, NBP3-47217), GAPDH (Zhuangzhibio, Xi’an, China, NC021). Signals were developed with horseradish peroxidase-conjugated secondary antibodies (Abbkine, Atlanta, GA, USA), followed by an ECL kit (Zeta LIFE). The band intensity was calculated with a Tanon 5200 imager and quantified with ImageJ software (v2.16.0, National Institutes of Health, Bethesda, MD, USA). The intensity was normalized to GAPDH levels, expressed as relative fold change against the control.

### 2.6. Histological Study

For immunohistochemistry, brain cryosections (14 μm) were fixed with 4% PFA for 30 min (min) and processed for antigen retrieval with 10 mM sodium citrate (pH 6.0) at 90 °C for 10 min, then cooled down to RT. The sections were washed three times in 1× PBS for 10 min each time, blocked in blocking buffer for 1 h at RT, and then incubated with primary antibodies overnight at 4 °C. The next day, sections were incubated with the corresponding species-specific secondary antibodies at RT for 3 h. Finally, sections were counterstained with DAPI for 5 min, sealed with 50% glycerin, and examined using an Olympus FV3000 confocal microscope (Olympus Corporation, Tokyo, Japan).

Antibodies used in this study were as follows: SOX10 (Oasis Biofarm, Shanghai, China, OB-PGP001-01, 1:500), PDGFRα (Abcam, Cambridge, UK, ab61219, 1:500), CC1 (Sigma OP80, 1:200, St. Louis, MO, USA), MBP (Abcam ab7349, 1:500, Singapore). Secondary antibodies: donkey anti-guinea pig Alexa Fluor 647 (Jackson ImmunoResearch, West Grove, PA, USA, #706-605-148, 1:500); donkey anti-rat Alexa Fluor 594 (Invitrogen A21203, 1:1000), donkey anti-mouse Alexa Fluor 594 (Invitrogen A-21208 1:1000), and donkey anti-rat Alexa Fluor 488 (Invitrogen A-21208 1:1000).

### 2.7. Immunofluorescence Imaging and Quantitative Analysis

All immunofluorescence images used for quantitative analysis were directly acquired as single optical plane images at the mid-plane of tissue sections or cell culture samples. Briefly, the upper and lower Z boundaries of the samples were determined, and images were captured at the axial midpoint to standardize sampling. The Z-axis scanning step size was set to the minimal hardware step (0.8 µm/pix) to ensure optimal sampling. Identical imaging parameters were strictly maintained across control and *Pbx3* knockout groups, including the effective optical section thickness (~1.7 µm), laser power, PMT gain, offset, objective magnification, scanning speed, and XY pixel resolution. No automatic brightness, contrast, or gamma adjustment was applied during image capture. Cells were identified by concurrent detection of intact nuclear signal and matched cytoplasmic labeling within the single optical plane. Only nuclei with complete contours in this section were counted; partial nuclear profiles and nuclear debris were excluded.

Raw 16-bit uncompressed TIFF files were exported directly from the microscope system without any signal modification, background subtraction, intensity scaling, or interpolation processing prior to quantification. All quantitative measurements were performed in a double-blind manner and uniformly using ImageJ software, under identical threshold and analysis criteria, for all experimental groups to ensure apples-to-apples comparison. No post hoc modification or recalibration of quantification parameters was performed after data acquisition. Quantification from a single optical section reflects relative cell density between groups rather than the absolute total cell number within the sample.

For MBP immunofluorescence analysis, the corpus callosum and cerebral cortex were manually outlined as separate regions of interest (ROI), and the integrated density was measured after background subtraction using a non-specific area. The corrected total fluorescence (CTF) was calculated as: CTF = Integrated Density (ROI) − (Area of ROI × Mean fluorescence of background). For each animal, at least three non-adjacent sections were analyzed, and the average CTF value was used for statistical analysis. To enable valid pairwise comparisons between control and cKO genotypes within each single developmental time point, normalization was conducted independently for P14, P27, and P50. For each age group, the mean CTF value of littermate control mice was defined as a baseline of 1.0, and fluorescence signals from individual cKO mice were calculated as a fold change relative to their age-matched control counterparts. No quantitative or statistical comparisons were performed across different ages.

### 2.8. EdU (5-Ethynyl-2′-deoxyuridine) Labeling

For EdU labeling in vivo, mice were intraperitoneally injected with 25 mg/kg EdU at postnatal day 1 (P1), and euthanized 24 h later. For EdU labeling in vitro, 10 μM EdU was added to siRNA-transfected cultures, and cells were fixed 6 h later. EdU staining was performed following the manual (Beyotime, Shanghai, China, C0071L) and co-staining protocols were performed as described above.

### 2.9. TUNEL (Terminal Deoxynucleotidyl Transferase dUTP Nick End Labeling) Assay

Apoptotic cells were detected using the DeadEnd™ Fluorometric TUNEL System (Promega, Madison, WI, USA, TB235) according to the user manual. Briefly, P2 brain sections were permeabilized with proteinase K (10 μg/mL) for 8 min at room temperature, followed by incubation with the TUNEL reaction mixture for 60 min at 37 °C in a humidified chamber. Nuclei were counterstained with DAPI.

### 2.10. Statistical Analysis

All statistical analyses were performed in Prism v.9.0 (GraphPad Software, Inc., Erie, PA, USA) and SPSS v.21.0 (IBM Inc., Armonk, NY, USA). The normality test was performed using the Shapiro–Wilk test, and the homogeneity of variance test was performed using Levene’s test. All data that met normality and homogeneity of variance were compared using a Two-tailed unpaired *t*-test and One-way ANOVA. Data sets that were normally distributed but did not meet homogeneity of variance were compared using a Two-tailed unpaired Welch’s *t*-test. Data that did not meet normal distribution were analyzed with the Mann–Whitney U test or Friedman’s test. Numerical values were described as the mean ± SEM and presented as bar graphs. Significance was denoted as * *p* < 0.05, ** *p* < 0.01, *** *p* < 0.001, or **** *p* < 0.0001.

## 3. Results

### 3.1. Pbx3 Expression Is Dynamically Regulated During Oligodendrocyte Differentiation

**Figure 1:**
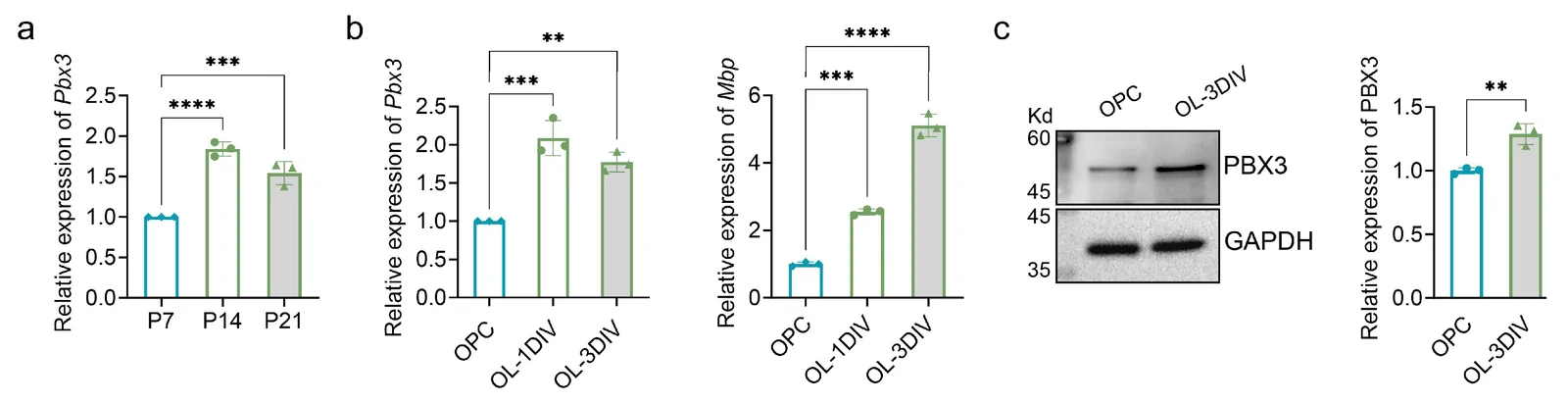
PBX3 Expression is Dynamically Regulated During Oligodendrocyte Differentiation. (**a**) Quantitative real-time PCR (qRT-PCR) analysis of *Pbx3* mRNA expression in corpus callosum tissues isolated from normal mice at postnatal day 7 (P7), P14, and P21. (**b**) qRT-PCR analysis of *Pbx3* and *Mbp* mRNA expression in primary OPCs and during in vitro differentiation at 1 day (OL-1DIV) and 3 days (OL-3DIV). (**c**) Western blot analysis of PBX3 protein levels in OPC and OL-3DIV. GAPDH was used as a loading control. Representative blots from three independent experiments are shown. The bar graph shows the quantification of PBX3 protein levels normalized to GAPDH. Data are presented as mean ± SEM. *n* = 3 mice per group in panel (**a**); *n* = 3 independent cultures in panel (**b**,**c**). ** *p* < 0.01, *** *p* < 0.001, **** *p* < 0.0001. One-way ANOVA followed by Dunnett’s multiple comparisons test was used in panels (**a**,**b**). Unpaired Student’s *t*-test was used in panel (**c**).

To investigate the potential role of PBX3 in oligodendrocyte development, we first examined its expression pattern during postnatal myelination and during oligodendrocyte differentiation in vitro. We isolated corpus callosum tissues from normal mice at postnatal day seven (P7), P14, and P21, and performed qRT-PCR analysis. P7 was used as the reference group. The results revealed that *Pbx3* mRNA levels were dynamically regulated during this period, with the highest expression observed at P14 (Figure 1a), a time point corresponding to the early phase of active myelination in the mouse corpus callosum.

To further characterize PBX3 expression at the cellular level, we employed a well-established in vitro differentiation system of primary OPCs. OPCs were cultured and induced to differentiate into oligodendrocytes. We collected samples at three distinct stages: undifferentiated OPCs, oligodendrocytes at day one in vitro (OL-1DIV), and oligodendrocytes at day three in vitro (OL-3DIV). OPC culture was used as the reference group. qRT-PCR analysis showed that *Pbx3* expression increased from OPCs to OL-1DIV, reaching the highest level at OL-1DIV, and then declined slightly at OL-3DIV (Figure 1b). As a control to confirm the differentiation status of the cultured cells, we also assessed the expression of *Myelin Basic Protein (Mbp)*, a well-established marker of mature oligodendrocytes. As expected, *Mbp* expression was minimal in OPCs and dramatically upregulated in OL-3DIV, confirming successful differentiation (Figure 1b).

To verify these findings at the protein level, we performed Western blot analysis on protein lysates from OPCs and OL-3DIV. Consistent with the mRNA data, PBX3 protein levels were significantly higher in differentiated OL-3DIV compared to undifferentiated OPCs (Figure 1c). We note that the in vitro system models oligodendrocyte differentiation rather than myelination per se, as it lacks axonal co-culture. Therefore, the temporal expression pattern observed in vitro reflects differentiation kinetics under simplified conditions and should not be directly equated with the in vivo myelination process. Nonetheless, these results demonstrate that PBX3 expression is positively correlated with oligodendrocyte differentiation, suggesting a potential role for this transcription factor in the differentiation process.

### 3.2. Pbx3 Deletion Impairs Oligodendrocyte Differentiation in a Developmental Stage-Dependent Manner

**Figure 2:**
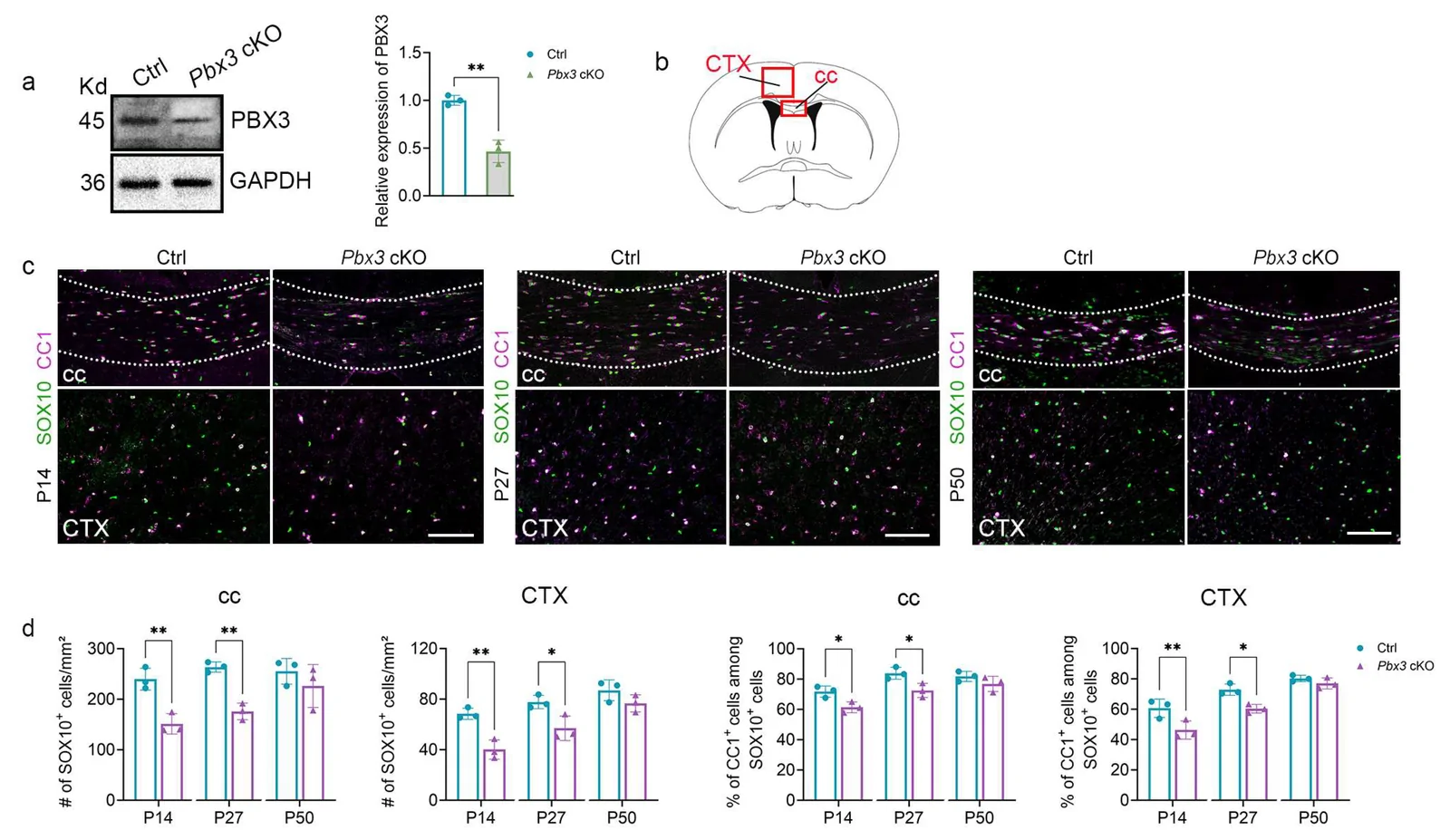
Oligodendrocyte-Specific *Pbx3* Deletion Impairs Oligodendrocyte Differentiation in a Developmental Stage-Dependent Manner. (**a**) Western blot analysis of PBX3 protein levels in the corpus callosum from control (Ctrl) and *Pbx3* cKO mice at P14. GAPDH was used as a loading control. Representative blots from three independent experiments are shown. The bar graph shows the quantification of PBX3 protein levels normalized to GAPDH. (**b**) Schematic diagram of a coronal brain section, with red boxes indicating the location of the corpus callosum (cc) and cortex (CTX) for image comparison between groups. (**c**) Representative immunofluorescence images of SOX10 and CC1 staining in the cc and CTX of control (Ctrl) and *Pbx3* cKO mice at P14, P27, and P50. Scale bar: 100 μm. (**d**) Quantification of SOX10-positive cell density and the percentage of CC1-positive mature oligodendrocytes in the cc and CTX of Ctrl and *Pbx3* cKO mice at P14, P27, and P50. Statistical analyses are limited to pairwise comparisons between control and cKO mice within each matched age; no statistical tests or direct quantitative comparisons are performed across different developmental ages. Data are presented as mean ± SEM. *n* = 3 mice per group. * *p* < 0.05, ** *p* < 0.01, Unpaired Student’s *t*-test was used.

To investigate the functional requirement of PBX3 in oligodendrocyte differentiation in vivo, we generated oligodendrocyte lineage-specific conditional knockout (cKO) mice. We crossed mice carrying a floxed allele of *Pbx3* (*Pbx3*^fl/fl^) with mice expressing Cre recombinase under the control of the *Olig1* promoter (*Olig1-Cre*), which drives Cre expression specifically in the oligodendrocyte lineage. The resulting *Pbx3*^fl/fl^; *Olig1-Cre*^+/−^ (hereafter referred to as *Pbx3* cKO) mice and their control littermates (*Olig1-Cre*^+/−^) were viable and fertile, allowing for subsequent developmental analysis. To confirm PBX3 protein reduction at the tissue level in *Pbx3* cKO mice, Western blot analysis was performed on protein lysates from the corpus callosum at P14. The results showed an approximately 50% reduction in PBX3 protein levels in cKO mice compared to controls (Figure 2a), indicating the successful recombination in the oligodendrocyte lineage. However, because this assay was performed on tissue lysates, it reflects overall protein reduction in the corpus callosum rather than deletion efficiency specifically in oligodendrocyte lineage cells.

We then examined the impact of *Pbx3* deletion on the oligodendrocyte lineage by immunofluorescence staining for SOX10, a transcription factor expressed throughout the oligodendrocyte lineage from OPCs to mature oligodendrocytes. Corpus callosum (cc) and cortical regions from P14 and P27 mice were selected for comparison (Figure 2b), since these two stages represent the beginning and peak of the myelination process, respectively. At both stages, the density of SOX10-positive cells was significantly reduced in *Pbx3* cKO mice compared to control littermates (Figure 2c,d). To assess whether this reduction reflected a defect in oligodendrocyte maturation, we quantified the percentage of CC1-positive mature oligodendrocytes among total SOX10-positive cells. In both the cc and cortical regions, the percentage of CC1-positive mature oligodendrocytes was significantly decreased in cKO mice at P14 and P27 (Figure 2c,d).

To determine whether the observed effects were transient or persisted into adulthood, we examined *Pbx3* cKO mice at P50, a time point when myelination is largely complete. Remarkably, both the density of SOX10-positive cells and the percentage of CC1-positive mature oligodendrocytes were comparable between control and cKO mice at this age (Figure 2c,d). These results indicate that PBX3 plays a role in regulating oligodendrocyte differentiation during the early postnatal period.

**Figure 3:**
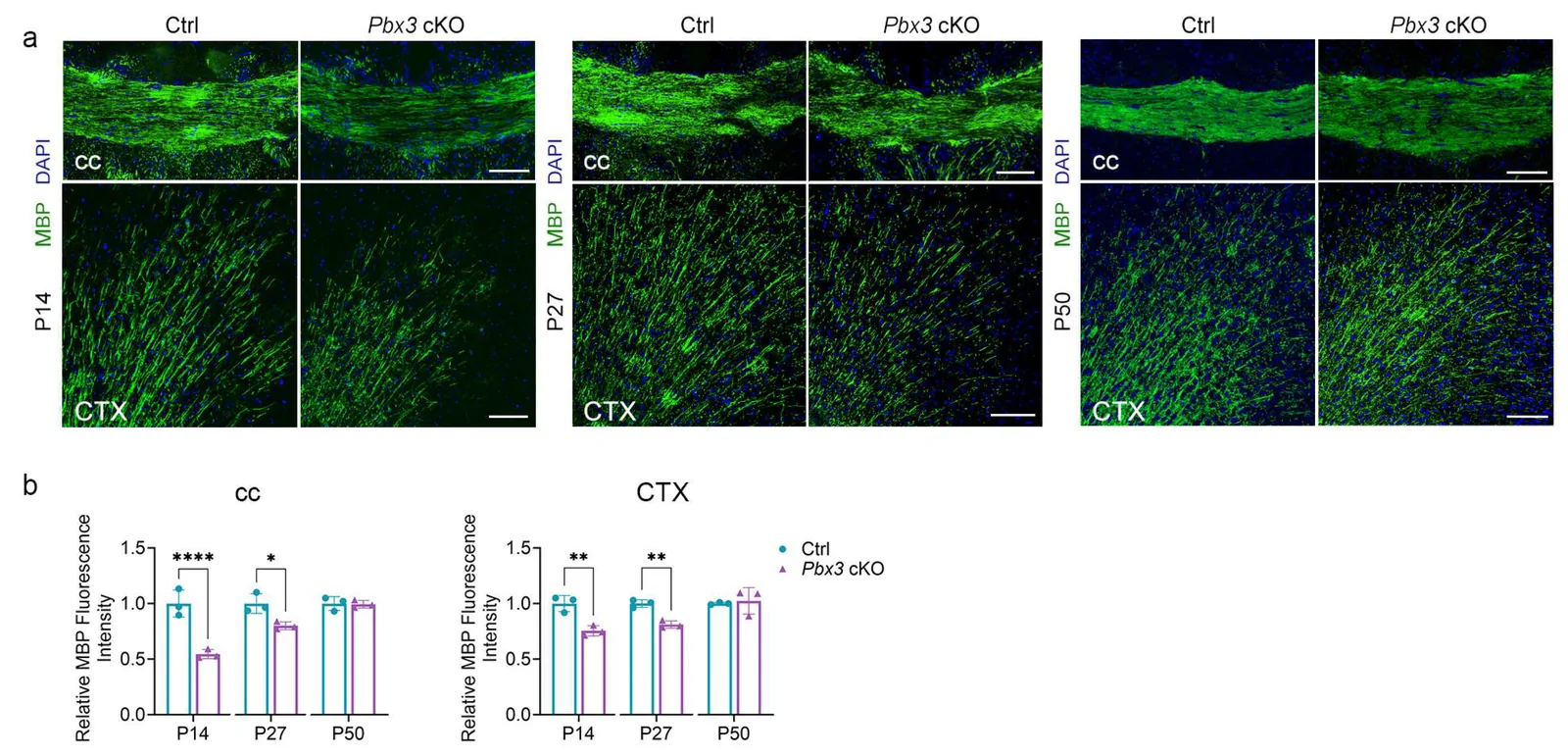
*Pbx3* Deletion Delays Myelin Gene Expression. (**a**) Representative immunofluorescence images of MBP staining in Ctrl and *Pbx3* cKO mice at indicated ages. Scale bar: 100 μm. (**b**) Quantification of MBP immunofluorescence intensity in cc and CTX of Ctrl and *Pbx3* cKO mice at P14, P27, and P50. Statistical analyses are limited to pairwise comparisons between control and cKO mice within each matched age; no statistical tests or direct quantitative comparisons are performed across different developmental ages. Data are presented as mean ± SEM. *n* = 3 mice per group. * *p* < 0.05, ** *p* < 0.01; **** *p* < 0.0001, Unpaired Student’s *t*-test.

We further evaluated myelination by immunofluorescence staining for MBP. Consistent with the mature oligodendrocyte analysis, MBP immunofluorescence intensity was significantly reduced in both the corpus callosum and cortex of *Pbx3* cKO mice at P14 and P27 (Figure 3). At P50, however, MBP intensity returned to control levels. These findings collectively suggest that PBX3 is important for proper oligodendrocyte differentiation and myelin formation during the early postnatal period, and its deficiency leads to a developmental delay that is largely recovered by adulthood.

### 3.3. Pbx3 Deficiency Suppresses OPC Proliferation In Vivo

**Figure 4:**
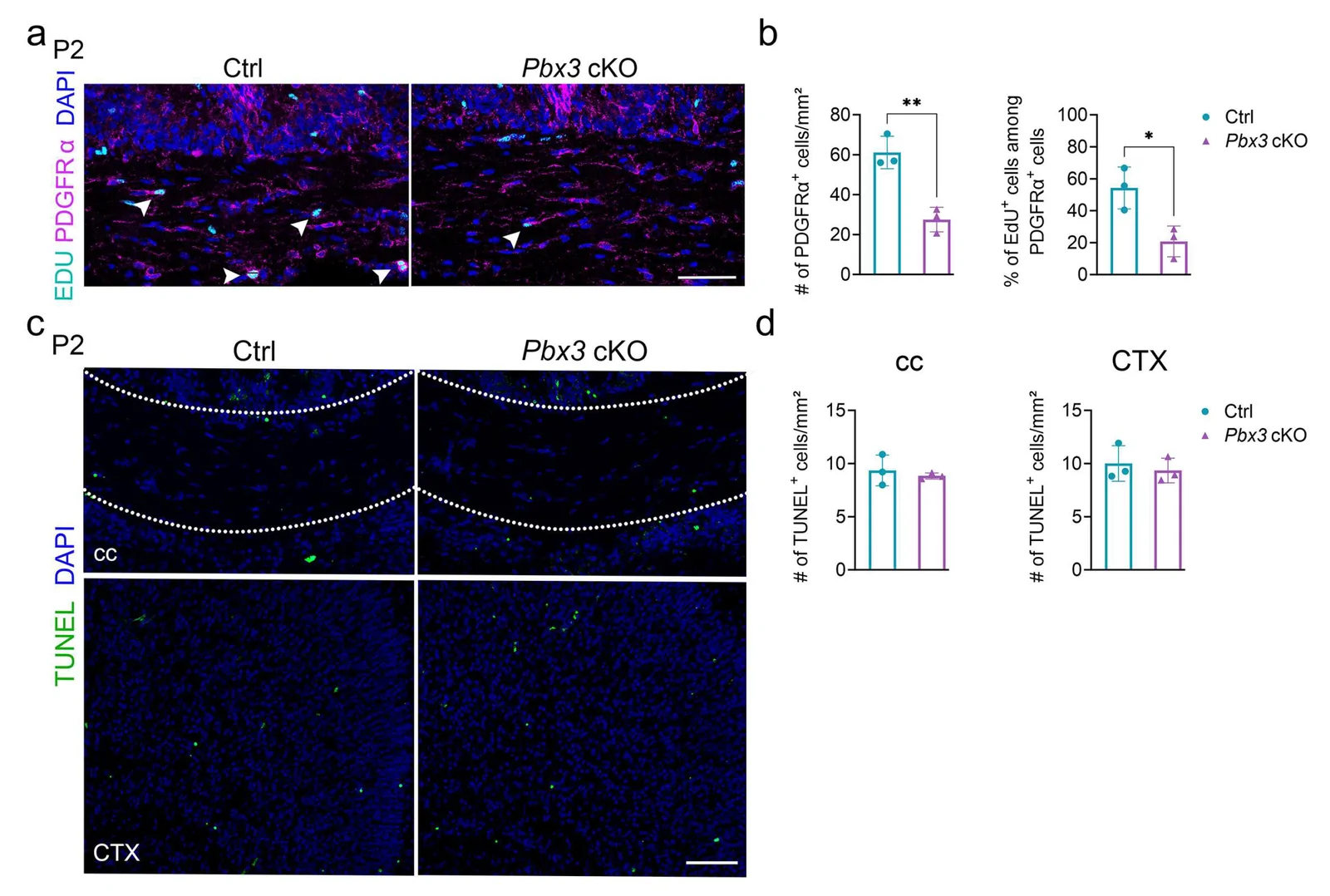
*Pbx3* Deficiency Suppresses OPC Proliferation In Vivo. (**a**) Representative immunofluorescence images of PDGFRα and EdU staining in the corpus callosum of control and *Pbx3* cKO mice. Arrows indicate PDGFRα-positive OPCs that are EdU-positive. Scale bar, 50 μm. (**b**) Quantification of the density of PDGFRα^+^ cells and the percentage of EdU^+^ cells among PDGFRα^+^ OPCs in the corpus callosum of control and *Pbx3* cKO mice. (**c**) Representative images of TUNEL staining in the brain of control and *Pbx3* cKO mice. Scale bar, 100 μm. (**d**) Quantification of the density of TUNEL^+^ cells in the cc and CTX of control and *Pbx3* cKO mice. Data are presented as mean ± SEM. *n* = 3 mice per group. * *p* < 0.05; ** *p* < 0.01, unpaired Student’s *t*-test.

Given the reduced density of SOX10-positive cells in the corpus callosum of *Pbx3* cKO mice at P14 and P27, we hypothesized that PBX3 might regulate the proliferation of OPCs. To test this, we performed EdU labeling experiments to assess OPC proliferation in vivo. P1 mouse pups were intraperitoneally injected with EdU, and the incorporation of EdU into PDGFRα-positive OPCs in the corpus callosum was analyzed 24 h later. Immunofluorescence staining for PDGFRα and EdU revealed a striking difference in OPC proliferation between control and *Pbx3* cKO mice (Figure 4a,b). We noticed that the density of PDGFRα^+^ cells was decreased in P2 *Pbx3* cKO mice, and in control animals, approximately 55.5% of PDGFRα-positive OPCs were EdU-positive, indicating active proliferation. In contrast, the percentage of EdU-positive OPCs in *Pbx3* cKO mice was dramatically reduced to approximately 20.8%. This result indicates that PBX3 might be involved in OPC proliferation during the early postnatal period. A key limitation of this observation is that the Olig1-Cre driver is active in early neural progenitors and embryonic OPCs, not exclusively in postnatal OPCs. Therefore, the in vivo phenotype cannot be attributed specifically to a cell-autonomous defect in postnatal OPCs, and we acknowledge that the reduced PDGFRα^+^ density at P2 could equally reflect reduced OPC generation/specification prior to or during early development. Future studies using temporally controlled inducible systems (e.g., Olig1-CreER or PDGFRα-CreER) will be necessary to determine the precise developmental stage at which PBX3 functions.

Nonetheless, the reduced OPC pool size, whether due to proliferation defects, impaired generation, or both, likely limits the number of cells available for subsequent differentiation and myelination during the early postnatal period.

To further examine PBX3 function in apoptosis, brain sections from P2 mice were subjected to a TUNEL assay. TUNEL staining revealed no significant difference in the number of positive cells in either the cc and or the CTX between control and *Pbx3* cKO mice (Figure 4c,d), suggesting that the reduced oligodendrocyte density is not primarily due to increased cell death.

Collectively, our findings establish that PBX3 is a positive regulator of early oligodendrocyte development, functioning to promote OPC proliferation and to support proper differentiation and myelin formation in a developmental stage-dependent manner.

### 3.4. Pbx3 Knockdown In Vitro Impairs OPC Proliferation and Oligodendrocyte Differentiation

**Figure 5:**
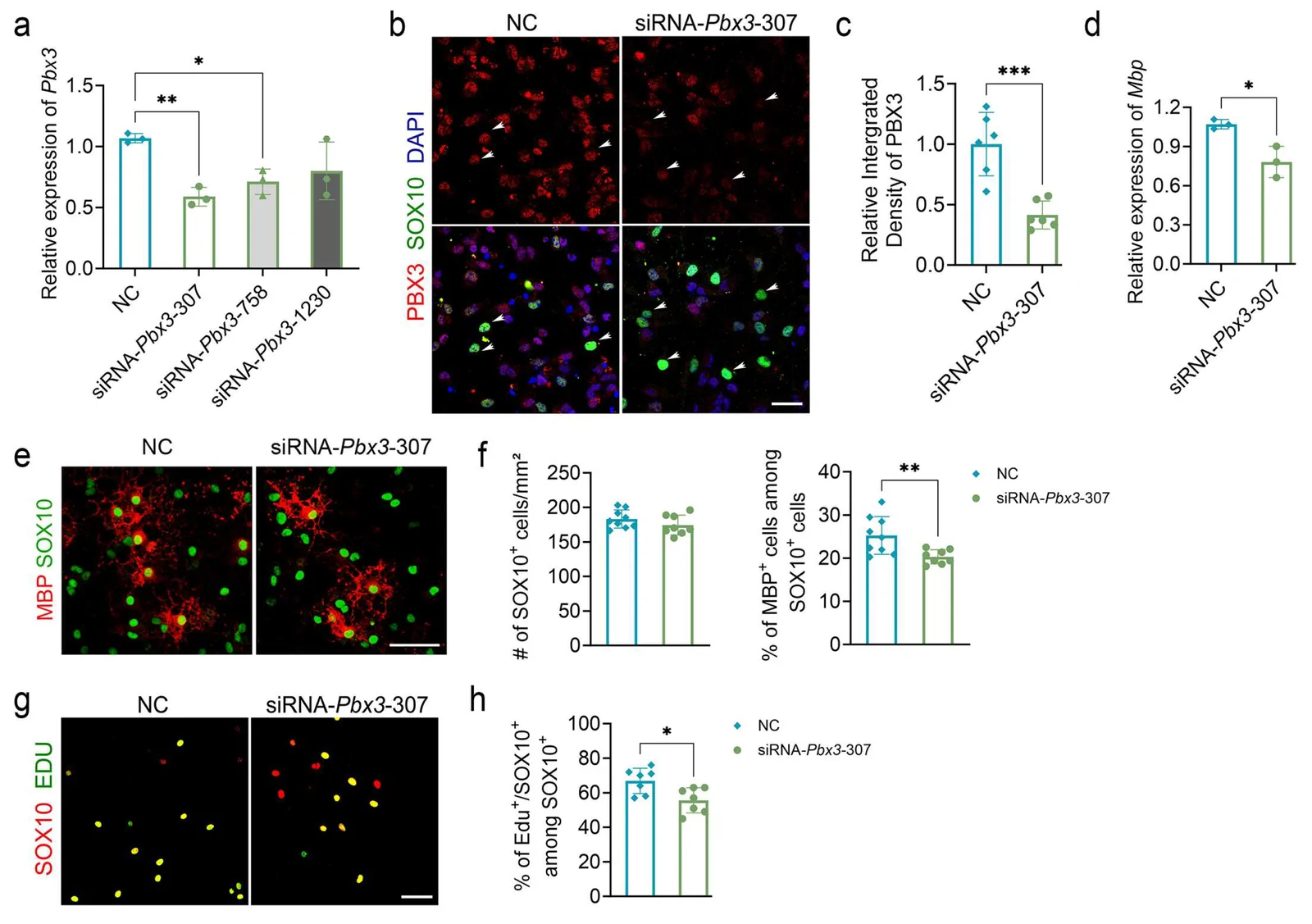
*Pbx3* Knockdown In Vitro Impairs OPC Proliferation and Oligodendrocyte Differentiation. (**a**) qRT-PCR analysis of *Pbx3* mRNA expression in primary OPCs transfected with NC or three independent siRNAs targeting *Pbx3*. (**b**) Representative immunostaining of PBX3 in siRNA-transfected cultures. Arrows indicate PBX3-positive cells that are SOX10-positive. Scale bar = 25 μm. (**c**) Quantification of the relative fluorescence intensity of PBX3 in SOX10-positive cells from two groups. (**d**) qRT-PCR analysis of *Mbp* mRNA expression in OPCs transfected with siRNA-*Pbx3*-307 and cultured under differentiation conditions for 72 h. (**e**) Representative immunofluorescence images of MBP and SOX10 staining in OPCs transfected with the indicated siRNA and differentiated for 72 h. Scale bar = 50 μm. (**f**) Quantification of the density of SOX10+ cells and the percentage of MBP-positive cells among SOX10-positive cells in transfected cultures. (**g**) Representative images of EdU incorporation assay in siRNA-transfected cultures. Scale bar = 50 μm. (**h**) Quantification of the percentage of EdU^+^ cells among SOX10^+^ cells. Data are presented as mean ± SEM. *n* = 3 independent experiments (in panels (**a**,**d**)), or *n* = 5–9 coverslips from 3 mice (in panels (**c**,**f**,**h**)). * *p* < 0.05, ** *p* < 0.01, *** *p* < 0.001; one-way ANOVA followed by Dunnett’s post hoc test in (**a**). Unpaired Student’s *t*-test in (**c**,**d**,**f**,**h**).

To further corroborate the in vivo findings, we performed siRNA-mediated knockdown of *Pbx3* in primary cultured OPCs. We designed three independent siRNAs targeting *Pbx3* and screened them for knockdown efficiency by qRT-PCR 48 h post-transfection. One siRNA (siRNA-*Pbx3*-307) effectively reduced *Pbx3* mRNA levels to approximately 58.8% of control levels (~41% reduction) and was therefore selected for all subsequent functional analyses (Figure 5a). An immunostaining assay confirmed reduced PBX3 fluorescence intensity in siRNA-*Pbx3*-307-transfected OPCs compared to control (Figure 5b,c). OPCs transfected with siRNA-*Pbx3-*307 or NC were cultured under differentiation conditions for 72 h. qRT-PCR analysis revealed a significant reduction in *Mbp* mRNA levels in siRNA-*Pbx3*-307-treated cells compared to controls (Figure 5d). Immunofluorescence staining further showed a marked decrease in the percentage of MBP-positive cells among SOX10^+^ cells (Figure 5e,f).

In addition, we tested EdU incorporation in siRNA-*Pbx3*-307-transfected cultures. Compared to control siRNA transfection, OPC cultures transfected with siRNA-*Pbx3*-307 showed a lower percentage of EdU^+^ cells among Sox10^+^ cells (Figure 5g,h), indicating impaired proliferation following *Pbx3* knockdown. Due to the technical limitations of plasmid transfection in primary OPC cultures, we were unable to perform rescue experiments with an siRNA-resistant *Pbx3* construct. Therefore, while the in vitro data support a role for PBX3 in OPC differentiation, they do not definitively establish cell-autonomy.

## 4. Discussion

In this study, we identified PBX3 as a positive regulator of oligodendrocyte differentiation during early postnatal development. Using a combination of in vivo and in vitro approaches, we demonstrated that *Pbx3* deficiency suppresses oligodendrocyte differentiation at multiple postnatal time points, and that *Pbx3* knockdown in cultured OPCs recapitulates this differentiation defect. Furthermore, we showed that PBX3 expression dynamically changes during distinct stages of oligodendrocyte lineage progression. These findings establish PBX3 as a novel player controlling oligodendrocyte differentiation and provide new insights into the molecular mechanisms underlying oligodendrocyte development.

The PBX family of TALE homeodomain transcription factors has long been recognized as essential co-factors for Hox proteins in regulating developmental processes [11]. The temporal expression pattern of PBX3 during oligodendrocyte differentiation—with dynamic changes across distinct stages—suggests that PBX3 may act at specific checkpoints in the oligodendrocyte lineage. This is consistent with the well-established concept that TALE proteins create a permissive chromatin platform that is selected by tissue-restricted transcription factors for binding [4]. In the context of oligodendrocyte differentiation, PBX3 may cooperate with oligodendrocyte lineage transcription factors such as OLIG1, OLIG2, SOX10, or MYRF to activate genes essential for differentiation [3,12].

The current study reports a transient requirement for PBX3 in oligodendrocyte development. While *Pbx3* deficiency caused significant deficits in oligodendrocyte differentiation and myelin gene expression at P14 and P27, these abnormalities were largely resolved by P50. This age-dependent phenotype indicates that *Pbx3* is not absolutely required for oligodendrocyte differentiation in general but rather plays a role during a specific temporal window of early postnatal development. The timing of this requirement (P14–P27) coincides with the peak period of active myelination in the mouse corpus callosum, suggesting that PBX3 may be particularly important for the rapid generation of mature oligodendrocytes during this developmental surge.

The recovery of oligodendrocyte differentiation and myelination by P50 in *Pbx3* cKO mice suggests that compensatory mechanisms can overcome the loss of *Pbx3*. To explore whether this recovery involves transcriptional upregulation of other PBX family members, we examined *Pbx1* and *Pbx2* mRNA expression in the corpus callosum of control and *Pbx3* cKO mice at P50 by qRT-PCR. Notably, we did not detect significant changes in their expression, indicating that the recovery is not mediated by increased transcription of these related family members. However, this does not exclude the possibility of compensation at the protein level—through increased protein stability, altered subcellular localization, or post-translational modifications—nor does it rule out functional redundancy among PBX family members that may be constitutively present. Alternatively, the observed recovery may simply reflect a developmental delay rather than an irreversible differentiation block, meaning that given sufficient time, oligodendrocyte differentiation can proceed even in the absence of *Pbx3*. Future studies examining PBX1 and PBX2 protein levels, as well as compound knockout models, will be required to fully elucidate the molecular mechanisms underlying the recovery phenotype.

TALE transcription factors, including PBX and MEIS family members, have emerged as critical regulators of neural development [13,14]. Our observation that PBX3 expression is involved in oligodendrocyte differentiation is particularly relevant in light of recent advances in understanding cooperative transcriptional regulation in oligodendrocytes. Choi and colleagues [15] recently developed coTF-reg, a single-cell multi-omics framework that systematically identifies cooperative transcription factor pairs in oligodendrocyte gene regulation. Using this approach, they demonstrated that key transcription factors such as SOX10, OLIG2, and MYRF extensively co-occupy oligodendrocyte-specific regulatory regions and function cooperatively to activate myelin gene expression. As a TALE homeodomain protein, PBX3 is well-positioned to participate in such cooperative regulatory interactions. TALE proteins are known to act as “co-factors” that facilitate the binding and function of other transcription factors rather than acting alone [4,16]. Therefore, we speculate that PBX3 may cooperate with one or more of these established oligodendrocyte key transcription factors—such as OLIG2, SOX10, or MYRF—to promote differentiation. The observation that *Pbx3* knockdown impairs MBP expression is consistent with this model, given that MBP is known to be co-regulated by SOX10 and NKX2.2 [15].

A recent study by Li and colleagues identified PBX3 as a significantly enriched gene in the context of ischemic brain injury, where it participates in the PBX3/Dguok/Kif21b signaling pathway that mitigates OPC dysfunction following middle cerebral artery occlusion [7]. While their study focused on the pathological response to ischemic injury, our findings extend this observation by demonstrating that PBX3 is also a positive regulator of OPC differentiation under physiological conditions. Collectively, these complementary studies suggest that PBX3 plays a dual role in OPC biology: supporting normal differentiation and development while also facilitating protective responses to injury.

Several other limitations of this study should be acknowledged. First, while we demonstrated that PBX3 is important for oligodendrocyte differentiation, the molecular mechanisms by which PBX3 exerts its effects remain unclear. Future studies using ChIP-seq to identify PBX3 genome-wide binding sites in OPCs and differentiating oligodendrocytes will be essential to define the direct transcriptional targets of PBX3. In addition, the identity of PBX3 interaction partners in oligodendrocytes—whether MEIS family members, HOX proteins, or other transcription factors—remains to be determined. Co-immunoprecipitation followed by mass spectrometry or proximity labeling approaches could address this question. Second, we did not perform a quantitative analysis of the sequential stages of oligodendrocyte differentiation, such as the transition from PDGFRα^+^ OPCs to CC1^+^ mature oligodendrocytes. Future studies using lineage tracing or stage-specific markers (e.g., ITPR2 for newly formed oligodendrocytes) will be needed to precisely define the differentiation stage at which PBX3 functions. Finally, in this study, we were unable to directly visualize PBX3 protein co-localization with oligodendrocyte lineage markers in tissue sections due to technical limitations with the available antibodies. However, qRT-PCR analysis of corpus callosum lysates showed developmentally regulated PBX3 expression, and Western blot analysis confirmed PBX3 protein expression in purified OPCs and differentiated oligodendrocytes. Future studies using RNAscope or newly generated antibodies will be needed to definitively determine the cellular distribution of PBX3 in the developing brain.

## Data Availability

The data supporting this study are available in the article, or available from the corresponding authors upon reasonable request.
